# Oxidation of Са^2+^-Binding Domain of NADPH Oxidase 5 (NOX5): Toward Understanding the Mechanism of Inactivation of NOX5 by ROS

**DOI:** 10.1371/journal.pone.0158726

**Published:** 2016-07-08

**Authors:** Irina Yu Petrushanko, Vladimir M. Lobachev, Alexey S. Kononikhin, Alexander A. Makarov, Francois Devred, Hervé Kovacic, Aslan A. Kubatiev, Philipp O. Tsvetkov

**Affiliations:** 1 Engelhardt Institute of Molecular Biology, Russian Academy of Sciences, Vavilov Street 32, 119991 Moscow, Russia; 2 Moscow Institute of Physics and Technology, 141700 Dolgoprudnyi, Moscow Region, Russia; 3 Aix-Marseille University, Inserm, CRO2 UMR_S 911, Faculté de Pharmacie, 13385 Marseille, France; 4 Institute of General Pathology and Pathophysiology, RAMS, 125315, Moscow, Russian Federation; Albany Medical College, UNITED STATES

## Abstract

NOX5 protein, one of the most active generators of reactive oxygen species (ROS), plays an important role in many processes, including regulation of cell growth, death and differentiation. Because of its central role in ROS generation, it needs to be tightly regulated to guarantee cellular homeostasis. Contrary to other members of NADPH-oxidases family, NOX5 has its own regulatory calcium-binding domain and thus could be activated directly by calcium ions. While several mechanisms of activation have been described, very little is known about the mechanisms that could prevent the overproduction of ROS by NOX5. In the present study using calorimetric methods and circular dichroism we found that oxidation of cysteine and methionine residues of NOX5 decreases binding of Ca^2+^ ions and perturbs both secondary and tertiary structure of protein. Our data strongly suggest that oxidation of calcium-binding domain of NOX5 could be implicated in its inactivation, serving as a possible defense mechanism against oxidative stress.

## Introduction

NOX5 protein from the family of NADPH-oxidases is one of the most active generators of reactive oxygen species (ROS), which plays an important role in numerous physiological and pathophysiological processes, such as regulation of cell growth, death and differentiation. NOX5 is expressed in reproductive systems, fetal organs [1, 2] as well as vascular tissue and is highly increased in cancer cell lines [3, 4]. It is involved in ROS-dependent signaling and regulation of transcriptional factors [2] and could form functional oligomers mediated by its dehydrogenase domain [5]. Disruption of expression level and regulation of NOX5 could be implicated in various diseases, such as atherosclerosis and endothelial dysfunction [6, 7]. Moreover, its deregulation could lead to transformation and uncontrolled growth of a number of cancer cells, including prostate cancer, adenocarcinoma [8], hairy cell leukemia, pancreatic and esophageal cancers [4, 6].

**Fig 1:**
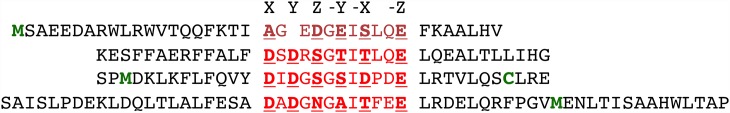
Sequence of the fragment 1–169 of Human NOX5 (isoform β) with four aligned EF-hand calcium-binding motives. Canonical EF-hand motives are shown in red, and non-canonical (with deletion at position Y) in brown. Amino acids which are involved in calcium binding (in positions 1, 3, 5, 7, 9 and 12 of motives) are underlined and are denoted by X, Y, Z, -Y, -X and -Z. Met1, Met 77, Cys107 and Met155 residues subjected to oxidation are shown in green.

NOX5 consists of six transmembrane domains and a long cytoplasmic C-terminus, which carries the binding sites of FAD and NADPH [2]. Unlike other NADPH oxidases, NOX5 has an extended N-terminus containing four EF-hand motifs (NOX5-EF) (Fig 1) and a consensus signal for calmodulin binding at the C-terminus. NOX5 possesses three canonical and one non-canonical EF-hand motifs [1, 9]. Calcium activation of NOX5 occurs in several stages: first, calcium ions bind to EF-hands of regulatory domain; second, conformational changes upon calcium binding lead to exposure of hydrophobic areas; and third, regulatory domain binds to catalytic domain in the C-terminus [9, 10] causing its activation. Thus, NOX5 can be activated either through its interaction with calcium-bound calmodulin [11] or directly by calcium [1]. To date, several mechanisms of NOX5 activation have been described. It was demonstrated that PKC-dependent phosphorylation of C-terminus of NOX5 β-isoform (NOX5β) increases its sensitivity to calcium, leading to NOX5 activation at lower level of intracellular calcium [12]. There is also a positive feedback mechanism, which consists of peroxide dependent activation of NOX5 by redox-dependent tyrosine kinase c-Abl [13]. Nevertheless, even though negative feedback is necessary to protect cells from NOX5 excessive activity, no inactivation mechanism of NOX5 has yet been reported.

In the present work, we characterized a direct mechanism of NOX5 inactivation by products of its catalysis, which oxidize the calcium-binding domain and block its activation. Indeed, using isothermal titration calorimetry, differential scanning calorimetry, and circular dichroism we found that oxidation of calcium binding domain of NOX5β perturbs both secondary and tertiary structure leading to a decrease of stoichiometry of this domain for calcium ions and to possible loss of its activation properties.

## Materials and Methods

### Materials

All chemicals (Sigma-Aldrich Co., USA) were of the highest grade. Recombinant NOX5-EF (residues 1 to 169 from NOX5 isoform beta plus a 8 residue long cloning stretch GSPGIHRD) was expressed and purified as described before [11]. The final purity was shown by electrophoresis in 15% acrylamide SDS gels. The protein concentration was measured spectrophotometrically using molar extinction coefficient at 278 nm of 18 053 M^-1^ cm^-1^ [14]. NOX5-EF was decalcified using TCA treatment prior to oxidation as described before [15].

### Isothermal Titration Calorimetry (ITC)

Binding of Ca^2+^ to NOX5-EF was analyzed by ITC using MicroCal VP-ITC instruments at 25°C in 50 mM Tris-HCl buffer, pH 7.5. NOX5-EF concentrations in the calorimetric cell ranged from 20 to 50 μM, whereas calcium concentrations in the syringe varied from 1 to 3 mM. The heat of dilution was measured by injecting calcium into the protein-free buffer solution; the value obtained was subtracted from the heat of reaction to obtain the true effective heat of binding. Data were analyzed using the MicroCal Origin software and were fitted with a “one set of sites” and led to the determination of affinity constants (*K*), stoichiometry (N) and enthalpy of binding (Δ*H*). Consequently, the entropy variations (Δ*S*) were calculated according to the standard equations. All experiments were repeated at least three times. The standard deviation of the determination of thermodynamic parameters did not exceed 10% for stoichiometry and enthalpy of binding and 20% for affinity constants.

### Differential Scanning Calorimetry (DSC)

Heat denaturation measurements were carried out on a MicroCal VP-DSC instrument in 0.51-ml cells at a heating rate of 1 Kmin^-1^. Experiments were performed in 50 mM Tris-HCl buffer in the presence of 1 mM EGTA or 1 mM CaCl_2_, pH 7.5. Protein concentration varied from 1.5 to 2.1 mg ml^-1^. Curves were corrected for the instrumental baseline obtained by heating the solvent used for protein solution. The reversibility of denaturation was checked routinely by sample reheating after cooling in the calorimetric cell. The denaturation parameters were determined assuming that the molecular mass of NOX5-EF is 19 812 Da and the partial specific volume is 0.72 cm^3^g^-1^. All experiments were repeated at least three times.

### Circular dichroism (CD)

CD spectra of NOX5-EF were recorded on a Jasco J-715 spectropolarimeter as described previously [16]. Far–UV CD experiments were performed in a 1-mm cell using protein solution with concentration 0.1–0.2 mg/mL. CD spectra were acquired in 50 mM Tris-HCl, pH 7.5. The results were expressed as molar ellipticity, [Θ] (deg cm^2^ dmol^-1^), based on a mean amino acid residue weight (MRW) of 114 Da for NOX5-EF. The molar ellipticity was determined as [Θ] = (θ×100MRW)/(*cl*), where *c* is the protein concentration in mg/mL, *l* is the light path in centimeters, and θ is the measured ellipticity in degrees. The secondary structure content was evaluated according to Johnson [17].

### NOX5-EF oxidation level analysis by LC-MS/MS

To reach full oxidation of NOX5-EF the protein was incubated during 2 hours in the presence of 50 mM peroxide hydrogen. These conditions are not meant to mimic oxidative environment in the cell wherein concentration of peroxide hydrogen probably do not reach such values, but they are necessary to fully oxidize the sample wherein protein concentration is also much higher than in cell. Such conditions are commonly used to get a reproducible sample preparation with oxidized methionine and cysteine residues *in vitro* [18–20]. To control the level of NOX5-EF oxidation, tandem mass spectrometry was used.

Protein was digested with trypsin gold mass spectrometry grade (Promega, Madisson, USA) according to the manufacturer’s protocol. The protein was diluted with 50 mM NH_4_HCO_3_ buffer (pH 8.1) and hydrolyzed with 0.005 mg/mL of trypsin at 38°C for 20 h. The concentration of the protein was 0.008 pmol/mL. The reaction was stopped with formic acid (up to 0.1% of acid in the sample). The resultant solution (hydrolysate) was analyzed using nano-flow LC-MS/MS as previously described [21]. Peptide mixtures were separated using an Agilent 1100 system autosampler/nanoHPLC (Agilent Technologies, Paolo Alto, CA, USA) and tandem mass spectrometry (MS/MS) analysis was performed on a 7-Tesla LTQ-FT Ultra (Thermo Electron, Bremen, Germany) mass spectrometer equipped with a nano-spray ion source (in-house system). Tryptic peptides were identified using Mascot (Matrix Science, London, UK; version 2.0.04) against the sequence of beta isoform of NOX5. The oxidation level of the protein was determined as intensity of oxidized tryptic peptides normalized to the summarized intensities of non-oxidized and oxidized peptides. Incubation with hydrogen peroxide (50 mM, 2h) leads to an increased proportion of oxidized Met1, Met 77, Met155 and Cys107 residues from 2–13% to 66–100%. Only fully oxidized samples were used for the study.

## Results

### NOX5-EF oxidation decreases the number of bound calcium ions

**Fig 2:**
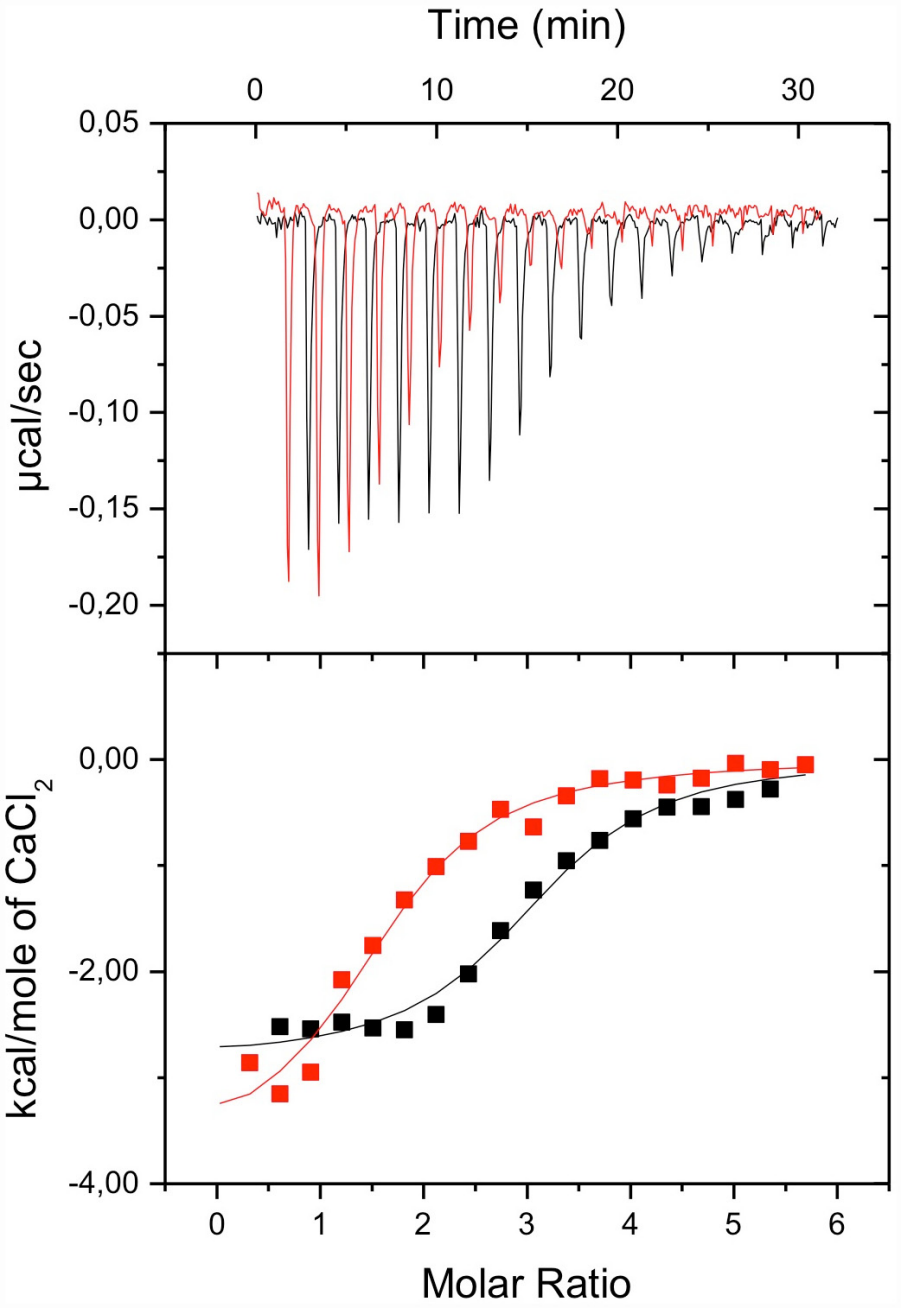
Typical ITC curves (upper panel) and binding isotherms (lower panel) of NOX5-EF interaction with CaCl_2_ with (red line) or without (black line) incubation with 50 mM H_2_O_2_ in 50mM Tris buffer (pH 7.5). All ITC titrations were performed in triplicate.

We used isothermal titration calorimetry (ITC) to investigate the influence of NOX5-EF oxidation on its interaction with Ca^2+^ ions. ITC data for Ca^2+^ binding to the native and oxidized NOX5-EF is shown in Fig 2; panel A presents raw calorimetric data for the ion-into-protein titration and panel B the binding isotherms obtained from raw calorimetric data by integrating each titration peak. Fitting of the binding isotherm of native NOX5-EF with the model of one-set-of-sites allowed us to determine all thermodynamic parameters of interaction and revealed the existence of three equal Ca^2+^-binding sites (which apparently corresponds to three canonical EF-hands) with association constants equal to (7.8±1.3)×10^5^ M^-1^. The chosen fit model implies that all binding sites are equal and bind calcium ions independently. Calcium binding to all sites is both enthalpy and entropy driven (ΔH = -14.2±0.9 kJ/mole; TΔS = 16.8 kJ/mole). This indicates a small favorable redistribution of the hydrogen bonding network upon calcium binding accompanied by a modest overall hydrophobic contribution [22, 23]. Thermodynamic parameters of interaction reflect the binding of calcium per se, *i*.*e*. the coordination of Ca^2+^ by EF-hands and the conformational changes of the protein. Oxidation of NOX5-EF in the presence of 50 mM H_2_O_2_ during two hours resulted in a drop in observed stoichiometry for calcium (which in the case of one-set-of-sites model corresponds to the inflection point of the binding isotherm) from three to two (Fig 2). However, the association constant and other thermodynamic parameters of calcium ions binding to NOX5-EF did not decrease significantly upon protein oxidation. Increasing the time of incubation of NOX5-EF in the presence of H_2_O_2_ to four hours did not lead to change of isotherm of calcium binding to oxidized NOX5-EF (data not shown). Thus, as a consequence of oxidation, NOX5-EF loses the ability to bind at least one calcium ion.

### Oxidation decreases NOX5-EF α-helical content and affects its tertiary structure

**Fig 3:**
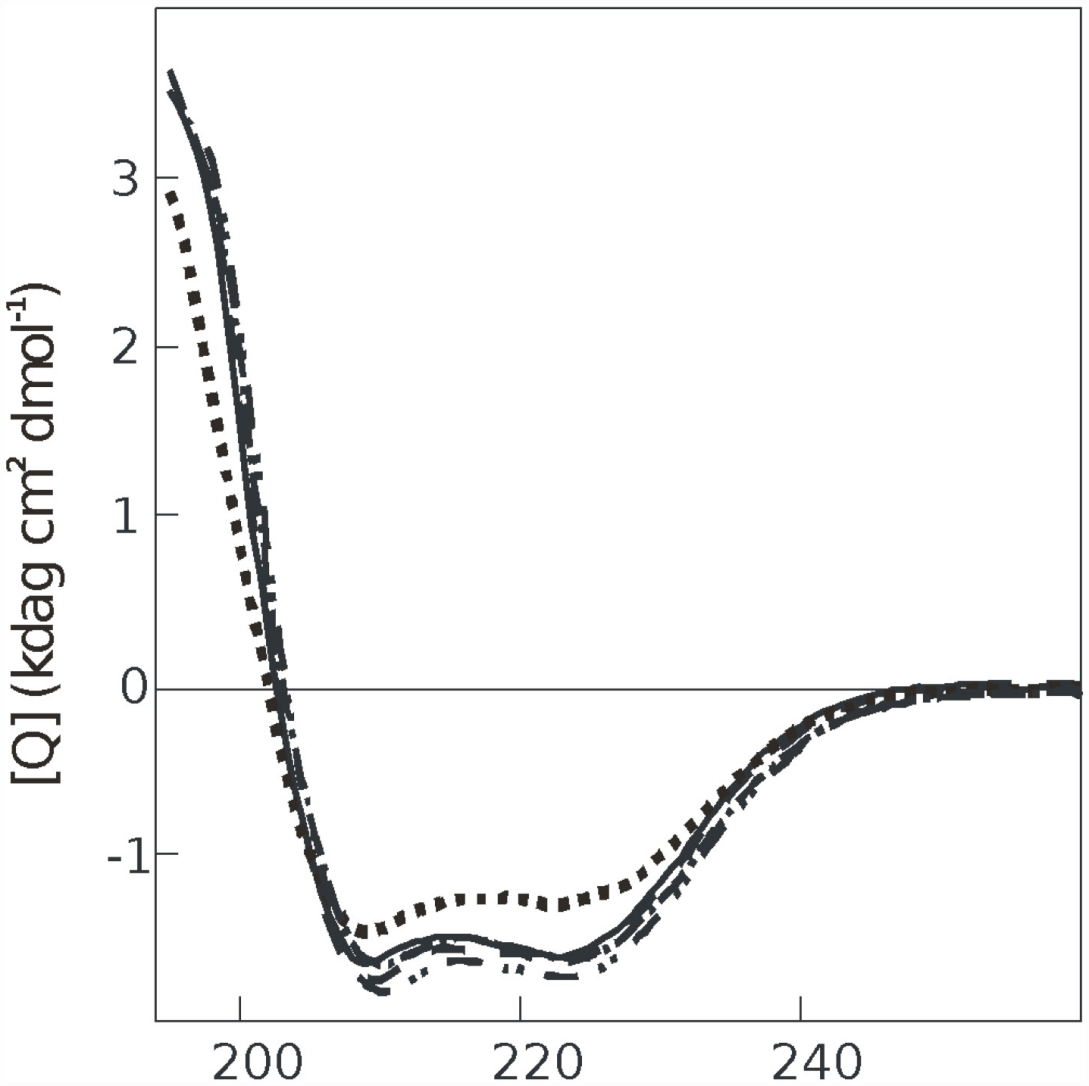
Far-UV CD spectra of NOX5-EF in the apoform in the presence of 50 mM H_2_O_2_ (dotted line); in the apoform, control (solid line); in the apoform in the presence of 1 mM CaCl_2_ and 50 mM H_2_O _2_ (chain line); in the apoform in the presence of 1 mM CaCl_2_ (dashed line) and in the apoform after incubation with 50 mM H_2_O _2_ in the presence of 1 mM CaCl_2_ (double chain line). All the CD spectra were acquired in 50 mM Tris-HCl, pH 7.5 at 20°C. All CD spectra were performed in triplicate.

To determine how oxidation affects secondary structure of NOX5-EF we used UV CD-spectroscopy. The far UV CD spectra of native and oxidized NOX5-EF are characteristic of a helical type with minima near 208 and 222 nm. Fig 3 shows the far UV CD spectra of the Ca^2+^-saturated (1 mM Ca^2+^) and metal-free forms (1 mM EGTA) of native and oxidized NOX5-EF in 50 mM Tris-HCl buffer at pH 7.5. At 20°C, calcium-free native NOX5-EF showed α-helical content close to 44%. Addition of calcium ions did not lead to significant increase of this value (Fig 3). In contrast, oxidized NOX5-EF demonstrated a significant drop of α-helicity from 44% in native form to 35% in oxidized form. Importantly, in the presence of calcium, oxidized NOX5-EF partially recovered secondary structure with 41% α-helicity (Fig 3). Thus, only in the absence of calcium there is a significant difference in secondary structure of the native and oxidized EF-hand domain of NOX5.

**Fig 4:**
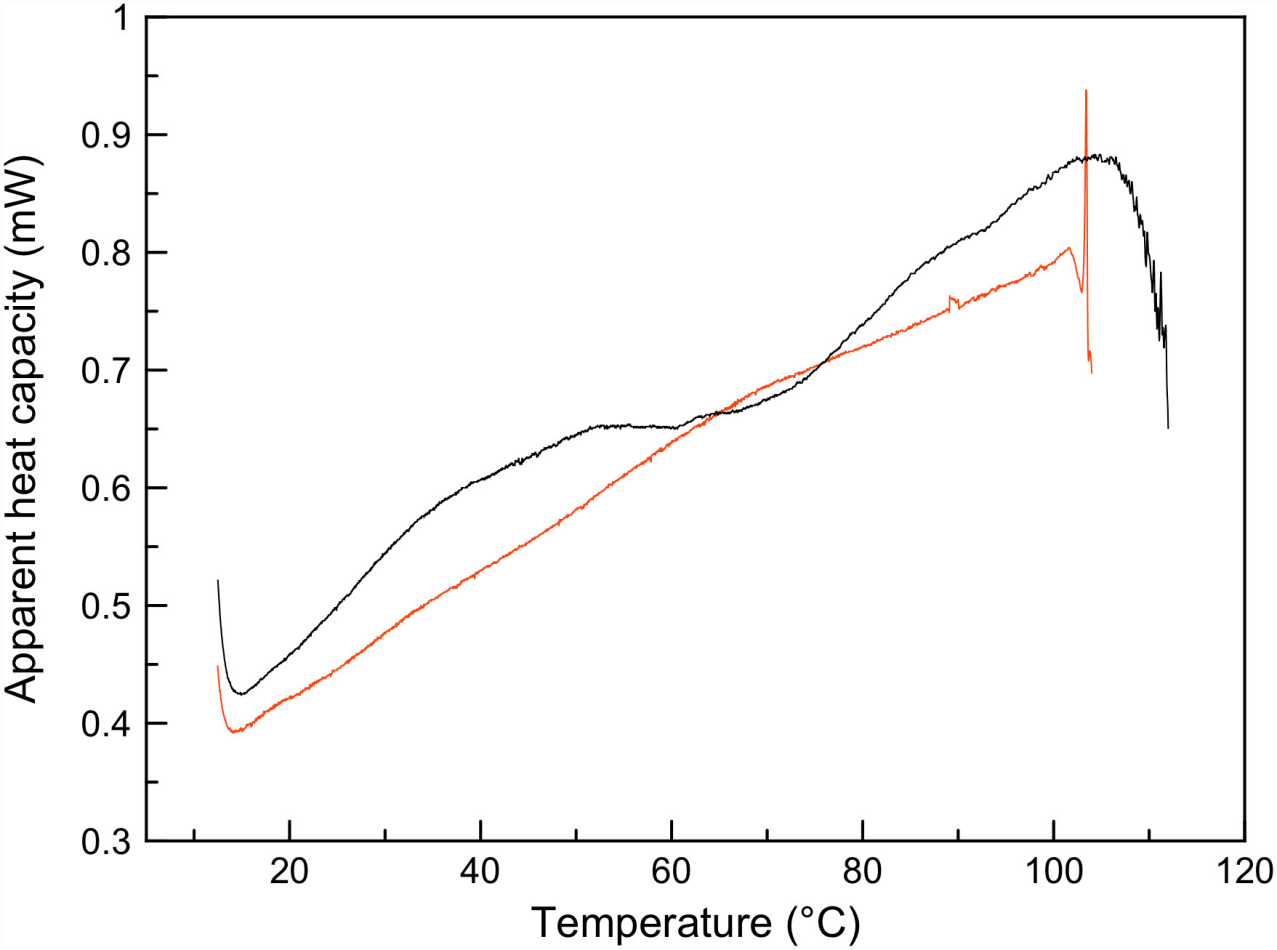
Temperature dependence of the heat capacity of native Ca^2+^-NOX5-EF (black line) and oxidized Ca^2+^-NOX5-EF (red line). All DSC melting were performed in triplicate.

To investigate if oxidation also affected the structure of the protein in the presence of calcium we used differential scanning calorimetry (DSC), which is particularly sensitive to changes in tertiary structure. Thermal denaturation of native NOX5-EF in the presence of calcium ions revealed the existence of several folding units (domains) (Fig 4) that unfolded at different temperatures [24, 25]. The first low-temperature peak between 25 and 60°C had two clear denaturation transitions, corresponding probably to the melting of two EF-hands. The second high-temperature peak could not be completely registered because of protein aggregation after 110°C. Nevertheless, this peak had a pronounced shoulder indicating the possible denaturation of two domains with melting temperatures about 90 and 105°C. Although it was not possible to deconvolute denaturation peaks because of protein aggregation at high temperatures, DSC data clearly demonstrated complex multi-domain organization of the native calcium-bound NOX5-EF. Oxidation of NOX5-EF caused the disappearance of all denaturation peaks and lead to protein aggregation already at 100°C. These data indicate that calcium binding does not restore NOX5-EF tertiary structure that was damaged by oxidation (Fig 4). In summary, CD shows that NOX5-EF secondary structure can be restored by Ca^2+^, while DSC shows that the loss of tertiary structure is permanent and cannot be rescued by Ca^2+^.

## Discussion

NOX5 plays an important role in a number of physiological and pathological processes via regulated generation of reactive oxygen species [1, 2, 6]. There are several activation mechanisms of NOX5 [6, 11, 13]. In contrast to other members of NADPH-oxidases family, NOX5 has its own regulatory calcium-binding domain and thus could be activated directly by calcium ions. When activated, NOX5 produce a significant amount of ROS. Recently it was demonstrated that peroxide ion radicals participate in positive feedback mechanism of NOX5 activation [13]. Such positive feedback should be constrained by the existence of inactivation mechanism of NOX5, which could protect cells from oxidative damage by products of NOX5 catalysis. Yet until now such mechanisms have not been elucidated. Still, recently it was demonstrated that the inactivation of NOX5 protects cells from radiation-induced DNA damage and cell death, which is in turn associated with oxidative stress [26].

**Fig 5:**
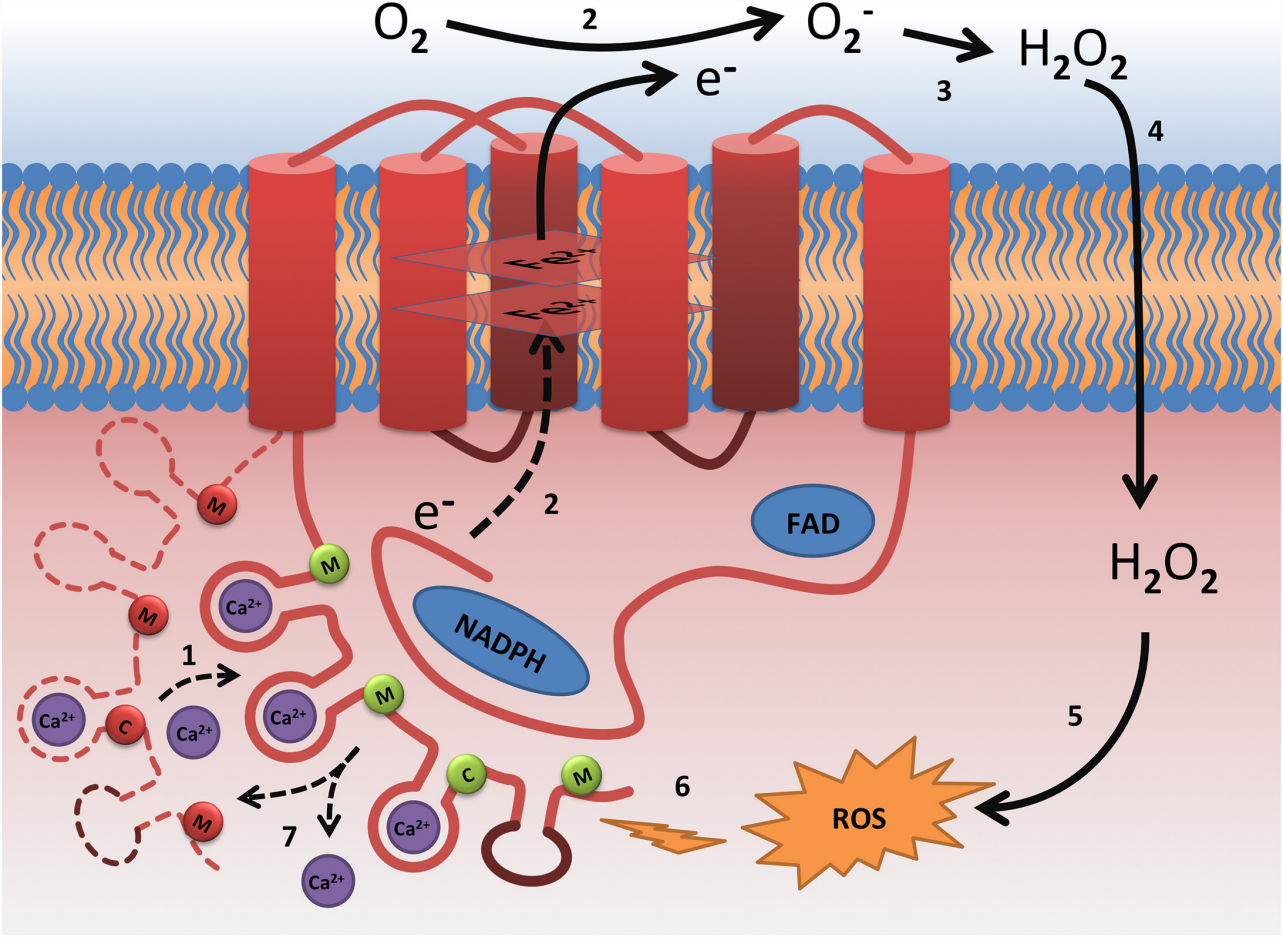
Model of NOX5 oxidative stress-mediated inactivation: 1) Ca^2+^ binding leads to conformational changes in the N-terminal EF-hand domain followed by its binding to catalytic C-terminal domain and NOX5 activation [9, 10]; 2) transfer of electrons from NADPH to oxygen [2, 3, 6]; 3) peroxide hydrogen formation [27]; 4) penetration of peroxide hydrogen through membrane into cell [27]; 5) ROS formation [27]; 6) oxidation of N-terminal domain of NOX5 by ROS; 7) this oxidation lead to loss of ability to bind all calcium ions necessary to activate catalytic domain. Canonical EF-hands are shown as red loops and non-canonical as brown loop. Methionine and cysteine residues marked with M and C letters respectively shown as circles (green for not oxidized and red for oxidized).

In the present work we investigated the impact of NOX5 calcium-binding domain oxidation on its structure and functional properties, by comparing structural properties of native and oxidized forms of EF-hand domain of NOX5. This domain contains three methionine and one cysteine residues, which can be oxidized. To obtain oxidized form of EF-hand domain of NOX5 we incubated native one in 50 mM peroxide hydrogen during 2 hours. These classical oxidation conditions are not meant to mimic oxidative environment in cell, but to obtain fully oxidized and reproducible protein samples, which is necessary for *in vitro* studies [18–20]. Using differential scanning calorimetry and UV CD-spectroscopy we demonstrated that oxidation perturbs both secondary and tertiary structure of NOX5-EF. Oxidative damage of the tertiary structure was greater than that of the secondary structure. Indeed, we observed only a 10% decrease of α-helicity upon NOX5-EF oxidation in calcium-free form that could be restored in the presence of calcium. On the contrary, tertiary structure of NOX5-EF was significantly damaged by oxidation and could not be restored even in the presence of calcium ions. Such an important perturbation of the tertiary structure of a protein with complex multi-domain organization should have a dramatic impact on its functional properties. The main functional property of NOX5-EF domain is the binding of calcium ions, which leads to conformational changes in the domain, its subsequent interaction with catalytic domain of NOX5 and its activation (Fig 5). As a result of catalytic activity of NOX5 the local concentration of hydrogen peroxide can increase significantly. Then peroxide hydrogen, which can easily penetrate through the membrane into the cell [27], can contribute to oxidation of methionine and/or cysteine residues of NOX5-EF. As we demonstrated, such oxidation provokes an important damage of the tertiary structure of NOX5 regulatory domain and decreases the number of calcium ions that bind to the protein and which are essential for NOX5 activation.

We have previously demonstrated that oxidation of methionine residues of calmodulin, which shares some structural and functional homology with NOX5-EF domain, results in perturbation of its secondary and tertiary structures [28, 29], and leads to a decrease of its stoichiometry for calcium and as a consequence loss of the ability to interact and activate some targets [28, 30]. Since calmodulin is involved in NOX5 calcium-dependent activation [11], it is possible that calmodulin oxidation might be also implicated in the mechanism of NOX5 inactivation. Furthermore, the existence of calcium-binding motifs (EF-hands) both in calmodulin [31] and NOX5-EF [9] as well as the presence of three methionine residues in the NOX5-EF sequence allowed us to suggest the direct mechanism of NOX5 inactivation by ROS.

NOX5 has four EF-hand motifs (three canonical and one non-canonical) composed of two alpha helical regions and 12-amino acid loop between them, coordinating calcium ions. Since two of the three methionine residues (Met79 and Met157) are located in the alpha helical regions of EF-hand motifs (Figs 1 and 5), it can be assumed that the oxidation of these methionine residues will distort structure of EF-hand motifs, resulting in one of them completely losing its ability to bind calcium. It should be noted that using ITC we registered the binding of only three calcium ions to NOX5-EF, which corresponds probably to binding on the canonical EF-hand motifs. The divergence between canonical and non-canonical EF-hands occurs in the N-terminal part of the binding loop, where Asp is replaced by Ala at position +X followed by a deletion at position +Y (Fig 1). Nevertheless, it was reported that non-canonical EF-hands can also coordinate calcium ions providing α carboxyl oxygen of Ala at position +X and water molecule oxygen at position +Y [9]. We could not observe the binding of fourth calcium ion to NOX5-EF by ITC probably due to the absence of structural changes, which leads to the absence of heat exchange. In this case we can hypothesize that the binding of three calcium ions to canonical EF-hands is sufficient for NOX5-EF binding to catalytic domain and NOX5 activation. This hypothesis is confirmed by an earlier study which demonstrates that the mutation in non-canonical EF-hand of NOX5 which blocks calcium binding does not affect calcium-dependent generation of reactive oxygen species [9].

Understanding the mechanisms of NOX5 inactivation is important to identify new NOX5 inhibitors. NADPH oxidases are involved in many pathologies and the search for NOX specific inhibitors represent an important goal. Identification of the NOX5 regulatory domain sensitive to oxidation in the present work opens the road for new specific NOX5 inhibitors identification. Screening of interactants of this domain according to the recently developed strategy [32] could lead to the discovery of important NOX5 inhibitors.

## Conclusions

In summary, we used differential scanning calorimetry and CD spectroscopy to demonstrate that oxidation of NOX5 regulatory domain leads to the loss of its secondary and tertiary structure. Using isothermal titration calorimetry we showed that this loss in turn leads to decrease of NOX5 stoichiometry for calcium ions, which are necessary for NOX5 protein activation. These data suggest that oxidation of NOX5 regulatory domain is implicated in direct inactivation mechanism by the products of catalysis of NOX5, which ultimately blocks the development of oxidative stress.

## Abbreviations

ITC: isothermal titration calorimetry
DSC: differential scanning calorimetry
CD: circular dichroism
MS: mass spectrometry
ROS: reactive oxygen species
NOX5-EF: domain 1–169 of NADH oxidase 5
